# Chrysin Ameliorates Cyclosporine-A-Induced Renal Fibrosis by Inhibiting TGF-β_1_-Induced Epithelial–Mesenchymal Transition

**DOI:** 10.3390/ijms221910252

**Published:** 2021-09-23

**Authors:** Rohan Reddy Nagavally, Siddharth Sunilkumar, Mumtaz Akhtar, Louis D. Trombetta, Sue M. Ford

**Affiliations:** 1 Department of Pharmaceutical Sciences, College of Pharmacy and Health Sciences, St. John’s University, Queens, NY 11439, USA; rohan.nagavally@gmail.com (R.R.N.); ssunilkumar@pennstatehealth.psu.edu (S.S.); akhtarm@stjohns.edu (M.A.); trombetl@stjohns.edu (L.D.T.); 2 Viatris Inc., 1000 Mylan Blvd, Canonsburg, PA 15317, USA; 3 Department of Cellular and Molecular Physiology, Penn State College of Medicine, Hershey, PA 17033, USA

**Keywords:** cyclosporine A, epithelial mesenchymal transition, chrysin, renal fibrosis, TGF-β_1_

## Abstract

Cyclosporine A (CsA) is a nephrotoxicant that causes fibrosis via induction of epithelial–mesenchymal transition (EMT). The flavonoid chrysin has been reported to have anti-fibrotic activity and inhibit signaling pathways that are activated during EMT. This study investigated the nephroprotective role of chrysin in the prevention of CsA-induced renal fibrosis and elucidated a mechanism of inhibition against CsA-induced EMT in proximal tubule cells. Treatment with chrysin prevented CsA-induced renal dysfunction in Sprague Dawley rats measured by blood urea nitrogen (BUN), serum creatinine and creatinine clearance. Chrysin inhibited CsA-induced tubulointerstitial fibrosis, characterized by reduced tubular damage and collagen deposition. In vitro, chrysin significantly inhibited EMT in LLC-PK_1_ cells, evidenced by inhibition of cell migration, decreased collagen expression, reduced presence of mesenchymal markers and elevated epithelial junction proteins. Furthermore, chrysin co-treatment diminished CsA-induced TGF-β_1_ signaling pathways, decreasing Smad 3 phosphorylation which lead to a subsequent reduction in Snail expression. Chrysin also inhibited activation of the Akt/ GSK-3β pathway. Inhibition of both pathways diminished the cytosolic accumulation of β-catenin, a known trigger for EMT. In conclusion, flavonoids such as chrysin offer protection against CsA-induced renal dysfunction and interstitial fibrosis. Chrysin was shown to inhibit CsA-induced TGF-β_1_-dependent EMT in proximal tubule cells by modulation of Smad-dependent and independent signaling pathways.

## 1. Introduction

Renal fibrosis is a common outcome of several forms of kidney injury. Irrespective of the etiology, chronic kidney disease (CKD) causes progressive decline in kidney function associated with loss of renal parenchyma and formation of scar tissue. Accumulation of scar tissue in the kidney leads to end-stage renal disease, requiring frequent dialysis or a transplant, both of which have been associated with complications such as infections or graft rejection. In spite of the treatment options available, patients suffering from CKD have a very high rate of mortality, making therapeutic intervention to inhibit renal fibrosis an urgent necessity [1].

Epithelial–mesenchymal transition (EMT) has been suggested as an important step in tissue repair following injury, through which the surviving epithelial cells transition into cells with a mesenchymal phenotype [2]. These cells migrate to the denuded basement membrane, proliferate to repair the injury [3,4] and finally re-differentiate to epithelial cells [5]. During EMT, renal proximal tubule cells lose epithelial characteristics, such as a cuboidal appearance and apical to basolateral polarity, and acquire mesenchymal traits including spindle shape, leading or trailing edge asymmetry and secretion of extracellular matrix proteins [6].

A key event in renal fibrosis is the initiation of EMT by TGF-β1, a cytokine produced by injured parenchymal cells and macrophages [5,7]. TGF-β1 elicits its effects on target genes, focusing primarily on Smad proteins and their role in the transcriptional regulation of extracellular matrix (ECM) genes (reviewed in [8]). Ligand activation of type 1 TGF-β1 receptor recognizes and phosphorylates the ligand-specific receptor-activated Smad, resulting in the formation of a heteromeric complex with co-Smad 4. Nuclear translocation of the complex induces transcription of various pro-ECM genes, including *Col1a1, Col3a1, and Timp1*, that eventually result in the development and progression of fibrosis. In addition, TGF-β1 can signal through Smad-independent signaling cascades (reviewed in [9,10]). The phosphoinositol 3-kinase (PI3K)-Akt-GSK3-β signaling axis is critical in the regulation of TGF-β1-induced EMT [11]. TGF-β1 triggers the PI3K/Akt/GSK-3β signaling cascade, resulting in the activation of Akt and subsequent inactivation of GSK-3β, which has been implicated as an early sequence in the induction of fibrosis [12,13,14]. Studies have also shown that phosphorylation of GSK-3β is a crucial step in triggering EMT [14,15]. Through these signaling cascades TGF-β1 regulates transcription and stabilization of DNA binding protein Snail [16,17,18]. The zinc finger transcription factor, Snail, functions as a potent repressor of E-cadherin expression that can, acting alone or in concert with the β-catenin, induce EMT [19,20,21].

Chrysin is a flavonoid (Figure S1) that can be extracted from natural sources such as passionflower (Passiflora caerulea, Passiflora incarnata) and honeycombs. This naturally occurring plant flavonoid has many pharmacological activities, which include anti-inflammatory [22], anti-apoptotic [23], antioxidant [24] and anti-cancer properties [25]. Chrysin has also been documented to counter drug-induced hepatotoxicity and nephrotoxicity (reviewed in Pingili et al., 2019 [26]). Chrysin has been shown to alleviate tubulointerstitial fibrosis [27] and glomerulonephritis [28] associated with diabetic kidney disease. The chronic use of the immunosuppressant cyclosporine A (CsA) results in impaired renal function as a result of fibrosis, which can have devastating effects on transplant patients. In the present investigation we evaluated the potential efficacy of chrysin in preventing CsA-induced renal fibrosis and elucidated a mechanism by which chrysin inhibits CsA-induced TGF-β1-mediated EMT in vitro.

## 2. Results

### 2.1. Effect of Chrysin on CsA-Induced Renal Dysfunction

**Figure 1:**
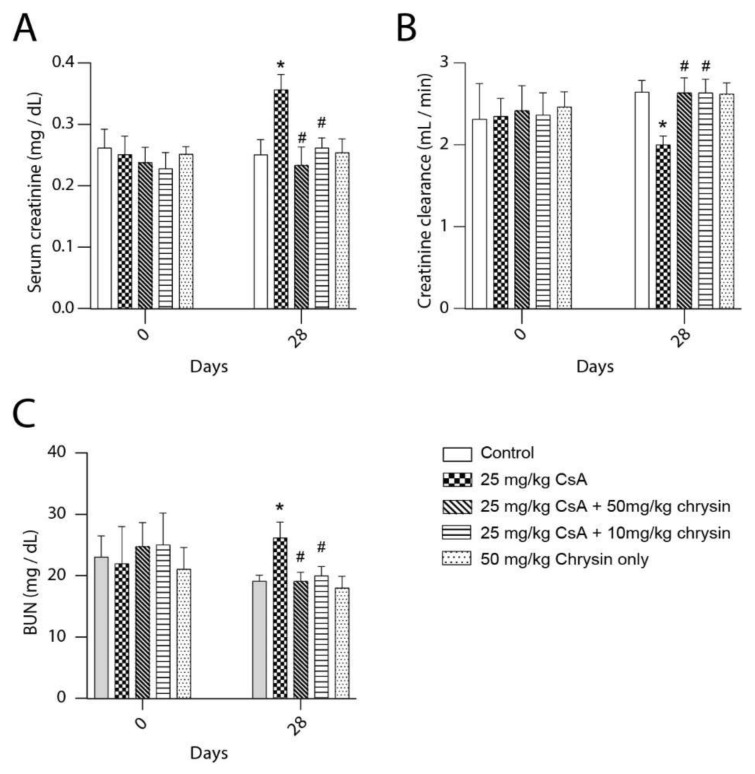
Effect of chrysin on cyclosporine A (CsA)-induced renal dysfunction. Male Sprague Dawley rats were treated with 25 mg/kg cyclosporine A (CsA) with or without 10 mg/kg or 50 mg/kg chrysin. (**A**) Serum creatinine, (**B**) creatinine clearance and (**C**) BUN levels measured on days 0 and 28 are shown. Data is represented as means ± SD with at least 5 animals per treatment group. Statistical significance was determined by one-way ANOVA followed by Tukey’s test for multiple comparisons. * indicates *p* < 0.05 versus control; # indicates *p* < 0.05 vs. CsA alone.

To determine the effect of CsA and chrysin on kidney function, serum and urine creatinine as well as blood urea nitrogen (BUN) levels were measured in samples collected from male Sprague Dawley rats. A significant elevation in serum creatinine was observed with CsA alone (Figure 1A); this was ameliorated by concomitant treatment with chrysin. Correspondingly, the creatinine clearance showed a significant decline after 28 days (Figure 1B), which was not seen in any of the animals co-treated with CsA and chrysin. Further chrysin co-treatment attenuated increased BUN levels seen with CsA treatment alone (Figure 1C).

### 2.2. Effect of Chrysin on Histopathological Damage Induced by CsA

**Figure 2:**
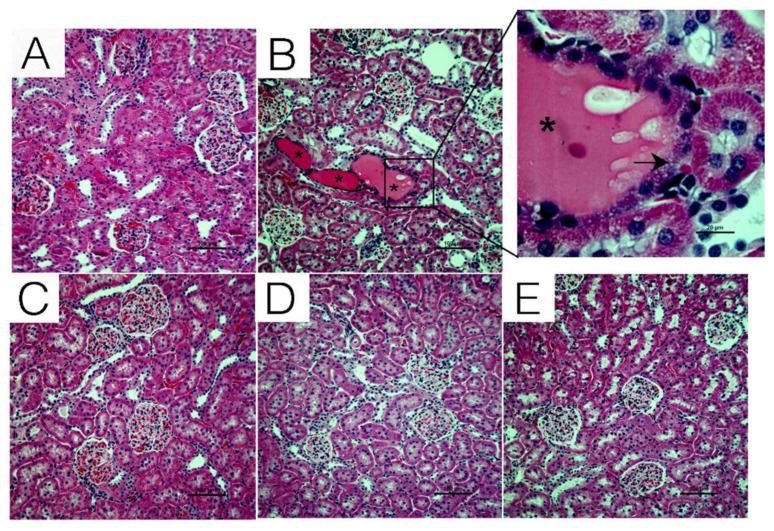
Histological evaluation of the effects of chrysin on CsA-induced tubular damage. Male Sprague Dawley rats were treated with CsA in combination with or without chrysin. Rats were euthanized on day 28; the kidneys were removed and fixed, and tubular damage was visualized using H&E staining. Compared to rats treated with (**A**) vehicle or (**C**) 50 mg/kg chrysin, tissue from animals treated with 25 mg/kg CsA (**B**) showed tubular dilation and edema. Appearance of casts (asterisk in the inset of B) and tubular vacuolization (black arrow in the inset) were also seen in kidneys from animals treated with CsA alone. Co-treatment with (**D**) 25 mg/kg CsA + 50 mg/kg chrysin or (**E**) 25 mg/kg CsA + 10 mg/kg chrysin showed no signs of tubular damage. Representative micrographs are shown at a magnification of 200× (inset 1000×). The scale bar represents 100 µm (panel (**B**) inset scale bar represents 20 µm).

Tubular damage induced by CsA treatment and the effects of co-treatment with chrysin were evaluated by H&E staining. Compared to control animals (Figure 2A), renal sections from animals treated with CsA alone (Figure 2B) showed evidence of tubular dilation and edema. There was also evidence of hyaline casts and tubular vacuolization (magnified in Figure 2B) in animals treated with CsA. Co-treatment with chrysin prevented these changes (Figure 2D,E).

### 2.3. Effect of Chrysin on CsA-Induced Collagen Deposition

**Figure 3:**
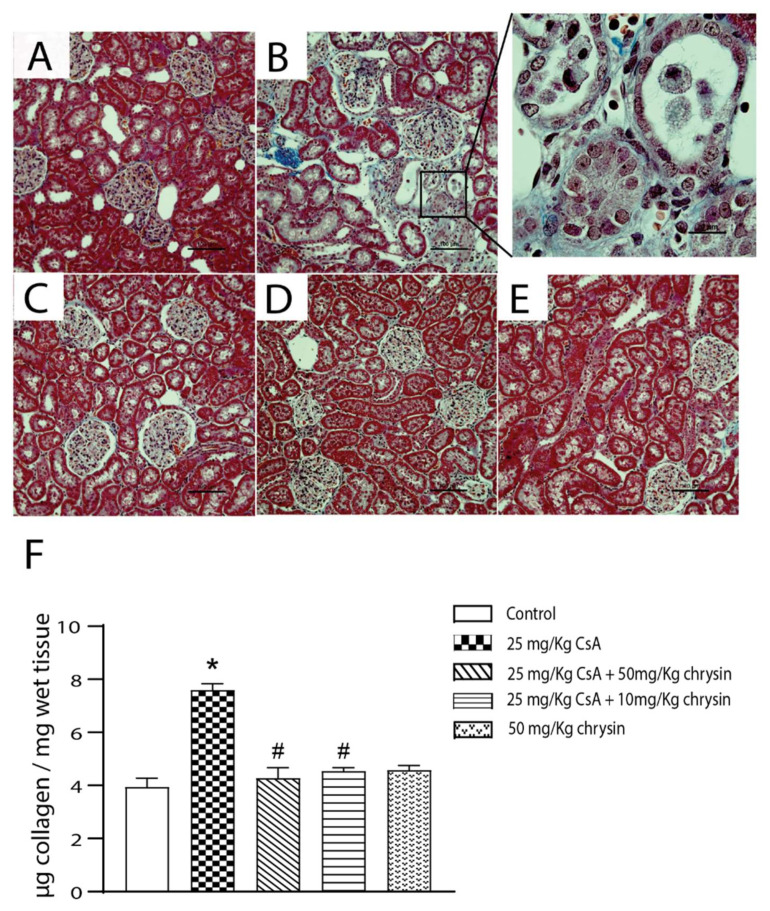
Effect of chrysin on CsA-induced collagen deposition. Male Sprague Dawley rats were treated with CsA in combination with or without chrysin. On day 28 the animals were euthanized; the kidneys were removed, fixed and stained for collagen (blue) using Masson’s trichrome. Letters indicate treatments: (**A**) control, (**B**) 25 mg/kg CsA, (**C**) 50 mg/kg chrysin, (**D**) 25 mg/kg CsA + 50 mg/kg chrysin and (**E**) 25 mg/kg CsA +10 mg/kg chrysin. Representative micrographs are shown at a magnification of 200× (magnified 1000×). The scale bar represents 100 µm. The magnified scale bar in (**B**) inset represents 20 µm. (**F**) Collagen content quantification in samples of the renal cortex was carried out using the Sircol Sirius red assay. The data are represented as mean ± SEM (*n* = 6). Statistical significance was determined by one-way ANOVA followed by Tukey’s test for multiple comparisons. * indicates *p* < 0.05 versus control; # indicates *p* < 0.05 vs. CsA alone.

The effect of chrysin on tubulointerstitial fibrosis induced by CsA was evaluated by Masson’s trichrome staining. Compared to vehicle-treated rats (Figure 3A), treatment with CsA (Figure 3B) resulted in increased deposition of collagen in the interstitium of cortical nephrons as evidenced by intense blue staining. Tissue sections from the animals co-treated with both CsA and chrysin did not show any evidence of elevated collagen deposition (Figure 3D,E). These histopathological observations were verified with quantification of collagen using a Sircol Sirius red assay (Figure 3F). Treatment with CsA alone significantly increased the collagen content in the kidney, which was prevented by co-treatment with chrysin.

### 2.4. Effect of Chrysin on TGF-β1- or CsA-Induced Changes in LLC-PK1 Cells

**Figure 4:**
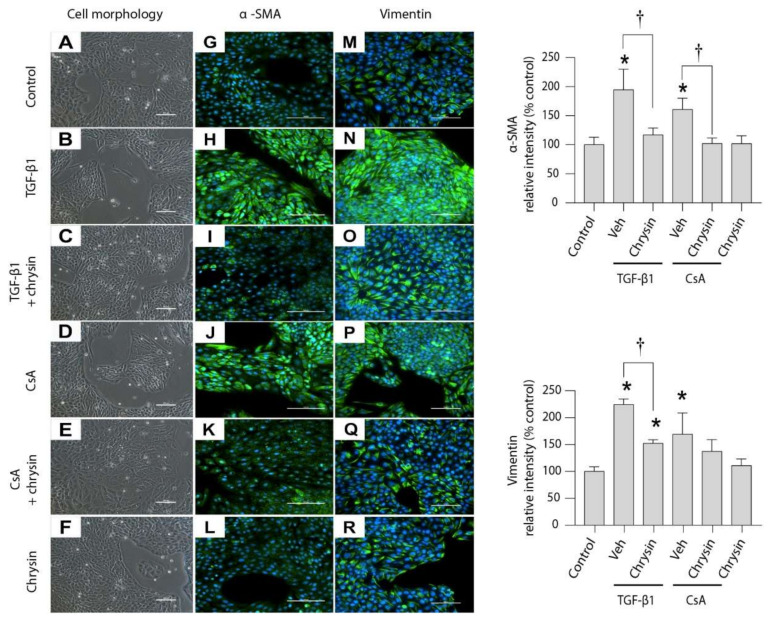
Effect of chrysin on TGF-β1- or CsA-induced EMT. LLC-PK_1_ cells were treated for 48 h with 5 ng/mL TGF-β1 or 4.2 µM CsA in the absence of 25 µM chrysin. Morphological changes (**A**–**F**) and immunofluorescence (green) of α-SMA (**G**–**L**) and vimentin (**M**–**R**) were assessed. Micrographs shown are representative of four individual experiments. For immunocytochemistry the nuclei were counterstained with DAPI (blue). The light micrographs were taken at a magnification of 200× (scale bar = 100 µm) and immunofluorescence micrographs were captured at a magnification of 400× (scale bar = 100 µm). Fluorescence for α-SMA and vimentin was quantified and data are represented as percent intensity relative to the control group. Statistical significance was determined by two-way ANOVA followed by Tukey’s multiple comparison test. * indicates *p* < 0.05 versus control; † indicates *p* < 0.05 versus chrysin co-treatment.

Treatment with either 5 ng/mL TGF-β1 or 4.2 µM CsA for 48 h induced alterations in LLC-PK1 cell morphology from an epithelioid-cobblestone-like appearance (Figure 4A) towards elongated, spindle-shaped cells (Figure 4B,D). Co-treatment with chrysin (Figure 4C,E) inhibited the change in morphology, retaining the epithelioid cobblestone appearance. Treatment with chrysin alone did not appear to change cellular morphology (Figure 4F). Immunofluorescence was used to evaluate the effect of chrysin on TGF-β1- or CsA-induced expression of mesenchymal markers. Compared to control cells (Figure 4G) or cells treated with chrysin alone (Figure 4L), treatment with TGF-β1 (Figure 4H) or CsA (Figure 4J) induced expression of α-SMA (green staining). Similarly, the expression of vimentin in cells treated with TGF-β1 (Figure 4N) or CsA (Figure 4P) was greater when compared against the basal level seen in controls (Figure 4M) and with chrysin only (Figure 4R). The induction in α-SMA (Figure 4I,K) and vimentin (Figure 4O,Q) by TGF-β1 or CsA was diminished on co-treatment with chrysin.

**Figure 5:**
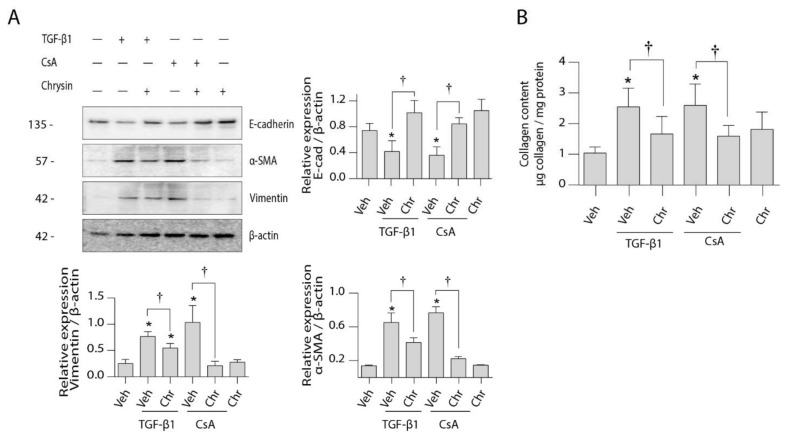
Effect of chrysin on CsA- or TGF-β1-induced EMT protein expression. LLC-PK1 cells were treated with vehicle (Veh), 4.2 µM CsA, 5 ng/mL TGF-β1 or 25 µM chrysin (Chr) or both for 48 h. (**A**) Protein expression of epithelial and mesenchymal markers were assayed using the Western blot method. Representative Western blots for E-cadherin, αSMA and vimentin are shown, and data are represented as mean intensity relative to β-actin ± SD of three individual experiments. (**B**) The amount of collagen deposited in the cell monolayers was assayed by Sircol™ Sirius red assay and normalized to protein content. Data are represented as mean collagen content ± SEM of four individual experiments. Statistical significance was determined by one-way ANOVA followed by Tukey’s multiple comparison test. * indicates *p* < 0.05 versus control; † indicates *p* < 0.05 versus chrysin co-treatment.

Protein expression analysis (Figure 5A) further showed that CsA or TGF-β1 treatment reduced epithelial E-cadherin expression while increasing expression of the mesenchymal markers α-SMA and vimentin. Chrysin co-treatment attenuated these effects. Chrysin treatment was also shown to decrease collagen deposition induced by TGF-β1 or CsA treatments (Figure 5B).

**Figure 6:**
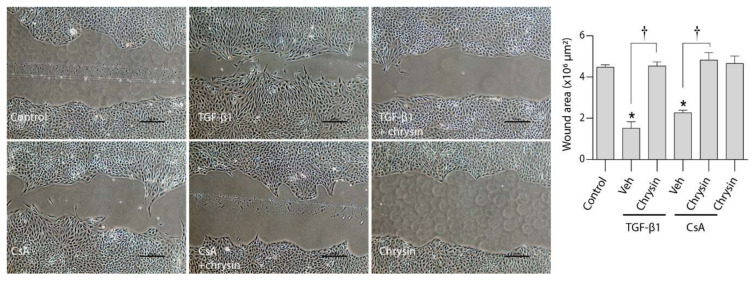
Effect of chrysin on CsA- or TGF-β1-induced cell migration. The effect of 25 µM chrysin on 5 ng/mL TGF-β1- or 4.2 µM CsA-induced LLC-PK_1_ cell migration was determined by a wound healing assay. Representative micrographs are shown, and data are represented as mean wound area ± SEM of three individual experiments. Statistical analysis was performed by one-way ANOVA followed by Tukey’s multiple comparison test. * indicates *p* < 0.05 versus control; † indicates *p* < 0.05 versus chrysin co-treatment. Veh—vehicle.

Furthermore, cell migration studies (Figure 6) showed that CsA or TGF-β1 treatments significantly enhanced cellular movement and wound healing. Co-treatment with chrysin prevented these alterations showing a similar wound area as the controls.

### 2.5. Effect of Chrysin on CsA-Induced TGF-β1 Signaling Cascade

**Figure 7:**
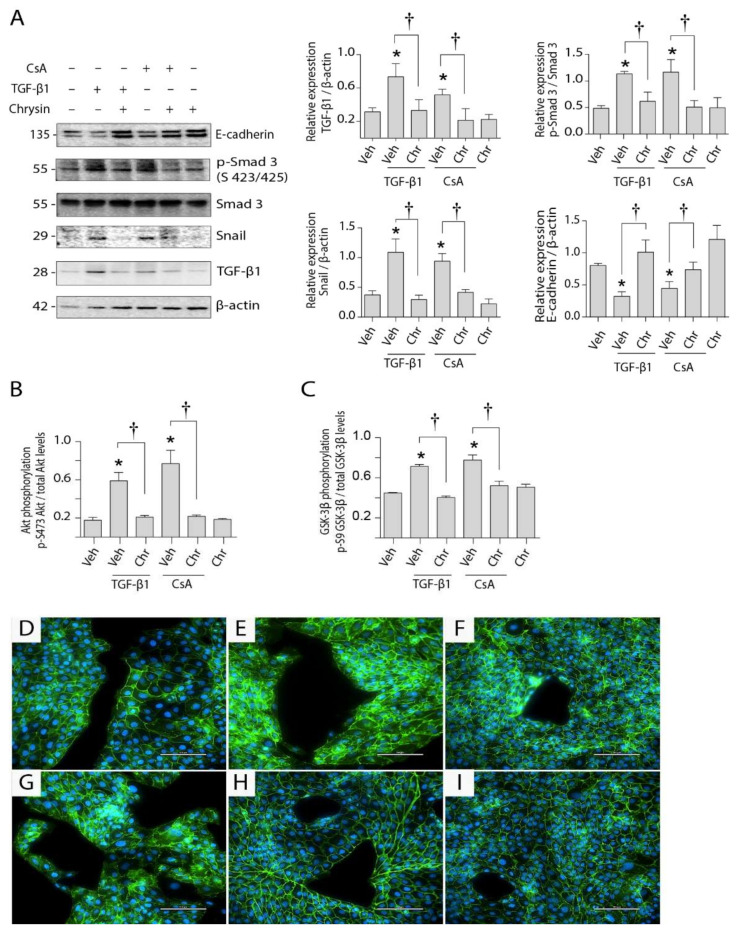
Effect of chrysin on CsA- or TGF-β1-induced EMT-signaling. LLC-PK1 cells were treated with vehicle (Veh), 4.2 µM CsA, 5 ng/mL TGF-β1 or 25 µM chrysin (Chr), or combinations. (**A**) Protein expression for TGF-β1, phospho-Smad3, total Smad 3 and Snail were determined by Western blot analysis. Phosphorylation of Akt (**B**) and GSK-3β (**C**) was determined by ELISA. Representative Western blots are shown, and data are represented as mean ± SEM of three individual experiments. Statistical significance was determined by one-way ANOVA followed by Tukey’s test for multiple comparisons. * indicates *p* < 0.05 vs. control; † indicates *p* < 0.05 vs. chrysin co-treatment. The effects of chrysin on CsA- or TGF-β1-induced localization of β-catenin (panels **D**–**I**) were also visualized. Release of membrane-bound β-catenin is indicated using white arrowheads. Treatments: (**D**) control, (**E**) 5 ng/mL TGF-β1, (**F**) 5 ng/mL TGF-β1 + 25 µM chrysin, (**G**) 4.2 µM CsA, (**H**) 4.2 µM CsA + 25 µM chrysin and (**I**) 25 µM chrysin. Scale bar indicates 100 µm.

Western blotting was used to determine the influence of chrysin on CsA-induced TGF-β1 signaling (Figure 7A). Treatment with 4.2 µM CsA increased the cellular expression of TGF-β1 whereas 5 ng/mL TGF-β1 treatment resulted in auto-induction of TGF-β1 expression. Co-treatment with chrysin prevented this observed increase. Downstream, CsA and TGF-β1 both induced phosphorylation of Smad 3 and increased expression of Snail and E-cadherin. Observations of TGF-β1-associated non-Smad pathways indicated that chrysin co-treatment decreased phosphorylation of Akt at the serine 473 position (Figure 7B) and of GSK-3β at the serine 9 position (Figure 7C), which were significantly increased after TGF-β1 or CsA treatments alone. The total Akt and GSK-3β levels were unchanged with the various treatments (data not shown). Additionally, intracellular localization of β-catenin was evaluated by immunofluorescence. The control cells had basal levels of β-catenin expression localized mostly to the cell–cell junctions (Figure 7D). Treatment with TGF-β1 (Figure 7E) or CsA (Figure 7G) increased localization of β-catenin within the cytoplasm (white arrowheads), whereas co-treatment with chrysin inhibited increases in the release of membrane-bound β-catenin (Figure 7F,H).

## 3. Discussion

Since the 1970s, cyclosporine A has revolutionized the field of organ transplants due to its immunosuppressant properties [29]. However, one of its major drawbacks has been chronic nephrotoxicity characterized by tubulointerstitial fibrosis [30,31]. Clinically, patients treated with CsA have noted loss of renal function accompanied by increased interstitial fibrosis and tubular atrophy [32,33,34]. Increased levels of TGF-β1 in the kidneys and blood have been documented in multiple types of renal fibrotic pathologies, including cyclosporine A nephropathy [35,36]. In vivo findings in rats treated with CsA [35,37] and in vitro studies using human proximal tubule cells [38,39] have demonstrated the role of EMT in CsA-induced renal fibrosis. Increased TGF-β1 secretion was observed followed by a decline in epithelial junction protein expression and increased mesenchymal characteristics [38]. Together, these studies suggest that targeting TGF-β1-mediated signaling pathways that cause EMT responses could be a potential therapeutic intervention against CsA-induced tubulointerstitial fibrosis. In the present investigation we show that co-treatment with the flavonoid chrysin offers nephroprotection against pharmacological induction of fibrosis by CsA treatment. We have further elucidated a potential mechanism by which chrysin prevents development of CsA-induced fibrosis by inhibiting TGF-β1-mediated EMT.

In this study, Sprague Dawley rats were used to investigate the potentially beneficial effects of chrysin on CsA-induced renal fibrosis. Treatment with CsA caused an increase in BUN and serum creatinine along with a decrease in creatinine clearance, markers for chronic CsA-induced nephropathy [31]. Similar to observations made by Kuruş et al. (2005) [40] and Ateşşahin et al. (2007) [41], kidneys from CsA-treated animals displayed classic signs of CsA nephrotoxicity, such as tubular dilatation, hyaline cast formation in the tubules and tubular vacuolization. Co-treatment with chrysin significantly improved renal function parameters and prevented CsA-induced tubular injury, demonstrating that chrysin offers protection against CsA-induced nephrotoxicity.

Fibrosis is a pathophysiological condition that occurs when chronic inflammatory stimuli that trigger wound healing lead to the excess deposition of extracellular matrix proteins [42]. Chrysin has been observed to attenuate fibrosis in numerous animal models of disease including asthma [43], carbon-tetrachloride-induced liver damage [44], adenine-induced chronic kidney disease [45] and diabetic nephropathy [27,28]. Studies on CsA-induced renal toxicity have shown increased collagen deposition indicative of tubulointerstitial fibrosis [46] as well as increased collagen content in cultured human renal epithelial (HK-2) cells [38], endothelial cells and fibroblasts [47]. In our investigation, chrysin co-treatment inhibited such CsA-induced increases in collagen deposition in the interstitium of rat renal cortices, as well as in cultured proximal tubule cells.

During organ fibrosis, type 2 EMT has been indicated as a mechanism responsible for reparative-associated processes in response to ongoing inflammation and oxidative stress [48]. A classic marker for EMT is change in cellular morphology [49] and cell motility [50], with increased expression of mesenchymal markers and decreased expression of epithelial proteins. Kang and coworkers (2015) [27] demonstrated the protective effects of chrysin against high-glucose-induced EMT in renal proximal tubule epithelial cells (RPTEC), characterized by reduced α-SMA and vimentin and increased E-cadherin expressions. Our observations indicate that CsA-induced morphological changes and expression of the mesenchymal markers, α-SMA and vimentin, are inhibited by co-treatment with chrysin. Chrysin co-treatment also prevented the corresponding decrease in E-cadherin expression. Chrysin has shown efficacy against cancer metastasis by decreasing in-cell motility via EMT inhibition [51]. Our findings reveal that CsA-induced cell migration is significantly decreased in cells co-exposed to chrysin. Both in vivo and in vitro observations point toward a novel role for chrysin in protection against CsA-induced fibrosis via inhibition of EMT.

**Figure 8:**
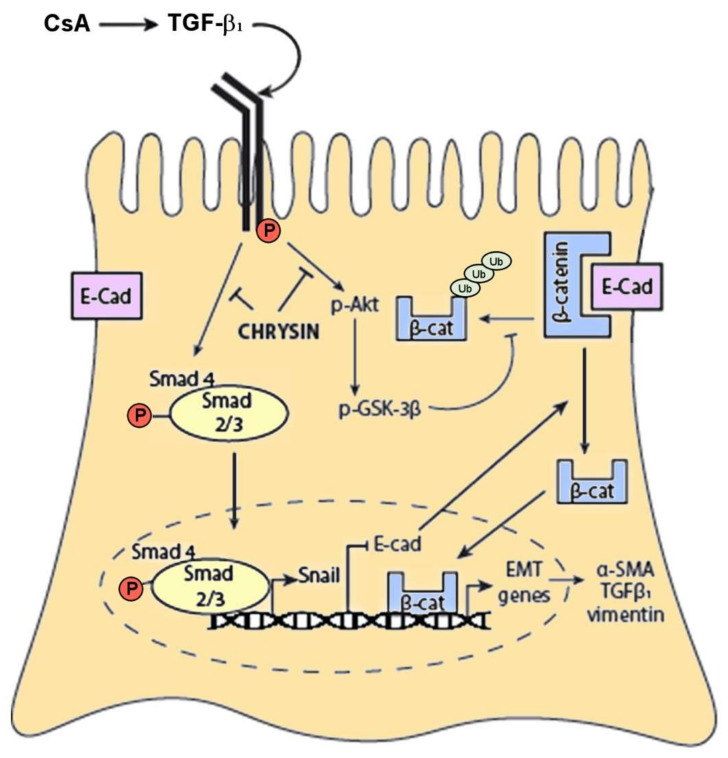
Mechanism of action of chrysin against TGF-β1-mediated EMT signaling chrysin inhibits CsA-induced TGF-β1/Smad-dependent increase in Snail expression, preventing a decrease in E-cadherin expression and subsequent localization of β-catenin into the cytoplasm. Chrysin also inhibits Akt/GSK-3β-mediated signaling, inducing the degradation of cytosolic β-catenin. Action on both of these pathways prevents cytosolic accumulation of β-catenin thereby inhibiting EMT and preventing the development and progression of fibrosis.

Studies to understand the molecular basis for anti-fibrotic actions of chrysin suggest that inhibition of CsA-induced EMT occurs by two separate mechanisms: (1) inhibition of TGF-β1/Smad 3 signaling cascade and (2) inhibition of TGF-β1-mediated Akt/GSK-3β signaling, both leading to a decrease in cytoplasmic localization of β-catenin (Figure 8). TGF-β1 phosphorylation of Smad 2 and Smad 3 has previously been linked to the initiation of EMT in multiple cancer cells [52] as well as proximal tubule cells [53]. CsA treatment for up to 24 h increased Smad 3 phosphorylation in rat glomerular mesangial cells [54]. Induction of the Smad pathway by TGF-β1 increases Snail expression [55], a known regulator of EMT [56,57]. Studies by Cano and coworkers (2000) have shown that TGF-β1 can trigger EMT by decreasing expression of epithelial junction proteins through the activation of Snail [56,58]. Downregulation of these proteins releases β-catenin initially immobilized at the adherens junctions [19], resulting in its nuclear translocation and initiation of EMT [59]. This correlates with the findings of the present study, showing that CsA-induced activation of the TGF-β1–Smad cascade led to increased phosphorylation of Smad3 and subsequent expression of Snail. Triggering of this cascade resulted in a decrease in E-cadherin expression leading to increased localization of β-catenin in the cytoplasm. β-catenin accumulation in the cytoplasm is critical to TGF-β1-induced EMT, and blocking its translocation has been shown to inhibit EMT [60]. The present study demonstrated that co-treatment with chrysin prevents this increase in cytoplasmic β-catenin by inhibiting the TGF-β1–Smad–Snail-mediated signaling.

TGF-β1 also acts via non-Smad pathways to mediate β-catenin release. Kang and coworkers (2007) [61]. have shown that TGF-β1 signaling activates the PI3K/Akt/GSK-3β signaling cascade, resulting in increased expression of the Ser-9-phosphorylated inactive form of GSK-3β. This prevents the ubiquitination and degradation of cytoplasmic β-catenin, thereby leading to its cytoplasmic accumulation and subsequent nuclear translocation [62,63]. We have shown that chrysin inhibits CsA-induced phosphorylation of Akt and the inactivation of GSK-3β preventing the cytoplasmic accumulation of β-catenin.

Agonists of PPAR-γ, such as telmisartan [64], troglitazone [18] and pioglitazone [65], have the ability to inhibit EMT. It is thus possible that chrysin may additionally act through a similar mechanism, as it is known to increase transcription and protein expression of PPAR-γ [18,66,67]. Increasing evidence of oxidative-stress-induced EMT has been documented in multiple pathologies including diabetic nephropathy [68], interstitial fibrosis of renal allograft [67], an in vitro model for chronic kidney disease [69] and carcinogenesis [70]. Antioxidant properties of chrysin [71,72,73] could be an additional avenue of protection against EMT. Chrysin has been shown to alleviate oxidant injury in lead-acetate-induced renal damage in rats [72]. Its antioxidant properties have also been useful in combating off-target effects of anticancer drugs such as mitomycin C [73]. Another molecule that has been implicated in the induction of EMT is HIF-1α [74,75]. The ability of chrysin to inhibit HIF-1α [76] and evidence of chrysin as a PPAR- γ agonist and antioxidant suggest that chrysin inhibition of CsA-induced EMT may involve additional mechanisms which require further investigation.

In summary, flavonoids such as chrysin offer protection against CsA-induced renal dysfunction and tubulointerstitial fibrosis. Chrysin prevents CsA-induced TGF-β1-mediated EMT in proximal tubule cells by inhibiting activation of the Akt/GSK-3β signaling pathway and Smad phosphorylation, leading to a decrease in cytoplasmic β-catenin localization. In conjunction with the in vitro study, the in vivo findings support the potential use of chrysin and its analogs as inhibitors of EMT to prevent fibrosis associated with cyclosporine A therapy.

## 4. Materials and Methods

### 4.1. Materials

Cells were grown in a customized formulation of SFFD (50:50 DMEM:Ham’s nutrient mix F12 with 15 mM HEPES) culture medium (GIBCO custom formula, F #00-5136 EL) purchased from GIBCO BRL (Grand Island, NY, USA). Culture media was supplemented with defined fetal bovine serum (FBS; Cat #SH30070) acquired from Hyclone Laboratories (GE Healthcare Life Sciences, South Logan, UT, USA). Human TGF-β1 was obtained from R&D Systems (Minneapolis, MN, USA; Cat #100-B-001). Chrysin was acquired from Acros Organics (Thermo Fisher Scientific, Somerset, NJ, USA; Cat #AC11032). Alfa Aesar was the vendor for cyclosporine A (Tewksbury, MA, USA; Cat #J63191). Collagen was determined with the Sircol™ Collagen Assay Kit (Accurate Chemical and Scientific, Westbury, NY, USA; Cat #S1000). Multispecies InstantOne™ ELISA kits to measure Akt (phospho/total) (Cat #85-86046-11) and GSK-3β (phospho/total) (Cat #85-86173-11) were purchased from Invitrogen (Carlsbad, CA, USA). Blood urea nitrogen (BUN) (Cat #2050) and creatinine (Cat #0430) were measured using kits from Stanbio Labs (Boerne, TX, USA). Antibodies used for Western blotting and immunofluorescence are listed in Table 1.

### 4.2. Animal Studies

Protocols involving the use of animals in these studies were submitted to and approved by the St. John’s University Institutional Animal Care and Use Committee (IACUC, protocol #1824, 8 August 2014). Eight-week-old male Sprague Dawley rats (Taconic, Germantown, NY, USA) weighing between 260–280 g were used for the study. To induce renal injury, cyclosporine A was administered subcutaneously at 25 mg/kg [77]. The intraperitoneal doses of chrysin, 10 and 50 mg/kg, were based on published studies [27,45,78]. The animals had access to food and water ad libitum for the duration of the study. Body weights were measured initially and at 3-day intervals.

Blood and urine samples were collected on days 0, 14 and 28. Blood samples were obtained from the lateral or ventral tail vein after placing the animals under isoflurane-induced anesthesia. Urine samples were collected using metabolism cages. The animals were acclimatized to the metabolism cages for 24 h and urine samples were collected over the next 24 h. The blood samples were analyzed for blood urea nitrogen (BUN) and creatinine, and urine samples were analyzed for creatinine content. Creatinine clearance was calculated by using the following formula:

$$\mathrm{Creatinine}\;\mathrm{clearence}=\frac{\mathrm{Urinary}\;\mathrm{creatinine}\;\left(\mathrm{mg}/\mathrm{mL}\right)\times \mathrm{Urine}\;\mathrm{volume}\;\left(\mathrm{mL}\right)}{\mathrm{Serum}\;\mathrm{creatinine}\;\left(\mathrm{mg}/\mathrm{mL}\right)\times \mathrm{Time}\;\left(\min\right)}$$

### 4.3. Analysis of Urine and Serum Chemistry

Creatinine in urine and serum in addition to serum BUN were measured with clinical reagent kits (Stanbio Labs, Boerne, TX, USA). Serum BUN (Cat #2020-500) was measured by a modification of the Berthelot urease method and creatinine (Cat #0430-500) by the creatinine amidohydrolase/sarcosine oxidase reactions. The methods were adapted for 96-well plate and absorbance was recorded at 550 nm using Promega GloMax Multiplus plate reader (Promega, Madison, WI, USA).

### 4.4. Histology

On day 28 animals were euthanized and kidneys were removed, transected and fixed in 10% neutral buffered formalin. The tissues were processed and embedded in paraffin. For morphological examinations, tissue sections of 4 μm thickness were stained with Gill’s hematoxylin and eosin–pholxine (H&E), whereas collagen localization was determined by staining tissue sections with Masson’s trichrome.

### 4.5. Cell Culture

LLC-PK1 clone 5 cells, isolated by Dr. S. Ford in 1990 from the established cell line, obtained from ATCC, were grown on 35 or 100 mm BD Falcon™ polystyrene tissue culture dishes (Thermo Fisher Scientific, Somerset, NJ, USA; Cat #353003). The cells maintain the epithelial morphology of the renal proximal tubule as well as characteristic SGLT transporters [79] and oxidative metabolism [80]. They were maintained in the custom culture medium reconstituted to the standard SFFD composition with a final glucose concentration of 5 mM, and supplemented with 3% FBS. The osmolality of the media was 317 mOsm/kg. Cells used in experiments were between passages 218 and 270 and were maintained at 37 °C in an atmosphere of 95% air to 5% CO_2_. The cultures were periodically checked for mycoplasma (Bionique Labs, Saranac Lake, NY, USA; Cat #M100).

### 4.6. Treatment of Cells

The cells seeded at a density of 2 × 10^4^ cells/mL were grown to about 60% confluence and then treated with fresh media containing CsA (4.2 µM), TGF-β1 (5 ng/mL), chrysin (25 µM) or combinations of chrysin with TGF-β1 or CsA. In vitro experiments were performed after 48 h of treatment. To determine phosphorylation of Smad 3, the cells were treated for 1 h before samples were collected.

### 4.7. Assessment of EMT in Vitro by Morphological Examination and Immunocytochemistry

LLC-PK1 cells were grown in the 4-well Nunc™ Lab-Tek II CC2™ chamber slide system (Thermo Fisher Scientific, Somerset, NJ, USA; Cat #154917) and treated for 48 h. After treatment, cell morphology was examined under the Nikon (Tokyo, Japan) Ts2R microscope and micrographs were captured. The cell monolayers were then fixed with 4% paraformaldehyde for 15 min at room temperature followed by permeabilization with 0.1% Triton X-100 for 10 min. Monolayers were blocked with 2% *w*/*v* bovine serum albumin (BSA) in PBS containing 0.3 M glycine for an hour and then incubated overnight at 4 °C with the primary mouse monoclonal antibodies against α-SMA (1:100), vimentin (1:500) or β-catenin (1:100). Monolayers were then washed with ice-cold PBS, incubated with FITC-labeled anti-mouse IgG antibody (1:1000) for 1 h, then washed thrice with PBS. After the final wash, the slides were mounted in Prolong™ diamond anti-fade reagent with DAPI (Thermo Fisher Scientific, Somerset, NJ, USA; Cat #P36962) and observed under a Nikon (Tokyo, Japan) Ts2R microscope.

### 4.8. Cell Migration Assay

Cell migration was quantified using an ImageJ macro for wound healing as described by Nunes and Dias (2017). In brief, LLC-PK1 cells were grown in 35 mm culture plates. After reaching 90% confluence, wounds ~2 mm wide were made in the monolayer with a 1 mL sterile pipette tip. Detached cells were removed by washing with 1 mL media and cells were then treated for 48 h. Micrographs were captured using the Nikon (Tokyo, Japan) Ts2R microscope and cell migration was evaluated using ImageJ software (Bethesda, MD, USA; ver. 1.50i).

### 4.9. Collagen Determination

Collagen content in cell and tissue lysates were determined with the Sircol™ Collagen Assay Kit (Accurate Chemical and Scientific, New York, NY, USA) as previously described [81]. In brief, collagen from samples of the renal cortex were collected and concentrated using acid pepsin digestion (0.1 mg/mL in 0.5 M acetic acid). Acid extracts were then incubated for 30 min with 0.1% Sirius red dye in picric acid at room temperature. The precipitated collagen–dye complex was pelleted by centrifuging at 12,000× *g* for 10 min. The supernatant was removed, and the pellet was washed with an acid/salt buffer and centrifuged again at 12,000× *g* for 10 min. The pellet was retained and solubilized with an alkali buffer and absorbance was measured at 560 nm.

### 4.10. Determination of Protein Expression by Western Blot Analysis

Cell lysates were obtained as described in [79,80]. In brief, after treatments cells were pelleted and lysed in RIPA buffer containing protease and phosphatase inhibitors for 30 min on ice, and centrifuged at 12,000× *g* at 4 °C for 15 min. Protein content was determined using the Bradford method; 25 µg proteins were separated using SDS-PAGE gradient gels (40–20% running gel; BioRad Cat #4561096) and electroblotted onto polyvinylidene fluoride (PVDF) membranes (Immobilon-P, Millipore-sigma; Merck Millipore Burlington, MA, USA; Cat #IPVF00010). The membranes were blocked with 2% BSA in Tris-buffered saline–Tween (TBST) buffer (0.1% Tween-20) for one hour and incubated overnight at 4 °C with the primary antibodies (Table 1). Membranes were then washed and incubated with appropriate secondary antibodies for 1 h. HRP-conjugated β-actin (1:5000) was used as a determinant of equal protein loading. After a series of washes in TBS–Tween buffer, protein bands were visualized by chemiluminescence with an ECL Prime luminescence kit (Amersham, UK) using an Alpha Innotech Fluorochem imager (ProteinSimple, San Leandro, CA, USA). Densitometry measurements were done using ImageJ software (Bethesda, MD, USA; ver. 1.50i).

### 4.11. Determination of Protein Expression by ELISA

LLC-PK1 cells treated for 48 h with TGF-β1 or CsA in the presence or absence of chrysin were lysed with RIPA buffer containing protease inhibitors. Lysates were centrifuged at 12,000× *g* for 15 min at 4 °C and supernatants were used. Phosphorylation of Akt and GSK was assayed using multispecies Instant One™ ELISA Kits for Akt (phospho/total) and GSK-3β (phospho/total) (Invitrogen, Carlsbad, CA, USA).

### 4.12. Statistics

Statistical analyses were conducted using the GraphPad Prism version 5 for Mac OS X (GraphPad Software, La Jolla, CA, USA; Apple Inc., Cupertino, CA, USA). All experiments were repeated at least four times. Data are expressed as mean ± SEM unless specified otherwise. Differences between the means were analyzed by one-way ANOVA followed by Tukey’s post hoc test. The criterion for statistical significance was set at *p* < 0.05.

## Figures and Tables

**Table 1:** List of antibodies.

Target	Catalog #	Dilution
Cell signaling technology		
E-cadherin	3195	1:1000
Smad 3	9523	1:1000
Phospho-Smad 3	9520	1:1000
Snail	3879	1:500
TGF-β_1_	3711	1:500
Santa Cruz Biotechnology		
β-catenin	sc-7963	1:1000
Vimentin	sc-6260	1:1000
Goat anti-rabbit IgG-HRP	sc-2004	1:10,000
Goat anti-mouse IgG-HRP	sc-2354	1:10,000
Invitrogen		
α-SMA	14-9760-80	1:1000
Anti-mouse IgG FITC	F2761	1:1000

## Data Availability

Not applicable.

## Supplementary Materials

The following are available online at https://www.mdpi.com/article/10.3390/ijms221910252/s1, Figure S1: Structure of chrysin.

## References

[1] Dalrymple L.S., Katz R., Kestenbaum B., Shlipak M.G., Sarnak M.J., Stehman-Breen C., Seliger S., Siscovick D., Newman A.B., Fried L. (2011). Chronic Kidney Disease and the Risk of End-Stage Renal Disease versus Death. J. Gen. Intern. Med..

[2] Grande M.T., Sánchez-Laorden B., López-Blau C., De Frutos C.A., Boutet A., Arévalo M., Rowe R.G., Weiss S.J., López-Novoa J.M., Nieto M.A. (2015). Snail1-Induced Partial Epithelial-to-Mesenchymal Transition Drives Renal Fibrosis in Mice and Can Be Targeted to Reverse Established Disease. Nat. Med..

[3] Humphreys B.D., Duffield J.D., Bonventre J.V. (2006). Renal Stem Cells in Recovery from Acute Kidney Injury. Minerva Urol. Nefrol. Ital. J. Urol. Nephrol..

[4] Ishibe S., Cantley L.G. (2008). Epithelial–Mesenchymal–Epithelial Cycling in Kidney Repair. Curr. Opin. Nephrol. Hypertens..

[5] Kalluri R., Weinberg R.A. (2009). The Basics of Epithelial-Mesenchymal Transition. J. Clin. Investig..

[6] Christiansen J.J., Rajasekaran A.K. (2006). Reassessing Epithelial to Mesenchymal Transition as a Prerequisite for Carcinoma Invasion and Metastasis. Cancer Res..

[7] Yamamoto T., Noble N.A., Miller D.E., Border W.A. (1994). Sustained Expression of TGF-Β1 Underlies Development of Progressive Kidney Fibrosis. Kidney Int..

[8] Verrecchia F., Mauviel A. (2002). Transforming Growth Factor-β Signaling Through the Smad Pathway: Role in Extracellular Matrix Gene Expression and Regulation. J. Investig. Dermatol..

[9] Derynck R., Zhang Y.E. (2003). Smad-Dependent and Smad-Independent Pathways in TGF-β Family Signalling. Nature.

[10] Rahimi R.A., Leof E.B. (2007). TGF-β Signaling: A Tale of Two Responses. J. Cell. Biochem..

[11] Medici D., Hay E.D., Goodenough D.A. (2006). Cooperation between Snail and LEF-1 Transcription Factors Is Essential for TGF-Β1-Induced Epithelial-Mesenchymal Transition. Mol. Biol. Cell.

[12] Kattla J.J., Carew R.M., Heljić M., Godson C., Brazil D.P. (2008). Protein Kinase B/Akt Activity Is Involved in Renal TGF-Β1-Driven Epithelial-Mesenchymal Transition in Vitro and in Vivo. Am. J. Physiol. Ren. Physiol..

[13] Curci C., Castellano G., Stasi A., Divella C., Loverre A., Gigante M., Simone S., Cariello M., Montinaro V., Lucarelli G. (2014). Endothelial-to-Mesenchymal Transition and Renal Fibrosis in Ischaemia/Reperfusion Injury Are Mediated by Complement Anaphylatoxins and Akt Pathway. Nephrol. Dial. Transplant..

[14] Du R., Xia L., Ning X., Liu L., Sun W., Huang C., Wang H., Sun S., Chernoff J. (2014). Hypoxia-Induced Bmi1 Promotes Renal Tubular Epithelial Cell–Mesenchymal Transition and Renal Fibrosis via PI3K/Akt Signal. Mol. Biol. Cell.

[15] Liang Y., Jing Z., Deng H., Li Z., Zhuang Z., Wang S., Wang Y. (2015). Soluble Epoxide Hydrolase Inhibition Ameliorates Proteinuria-Induced Epithelial-Mesenchymal Transition by Regulating the PI3K-Akt-GSK-3β Signaling Pathway. Biochem. Biophys. Res. Commun..

[16] Cho H.J., Baek K.E., Saika S., Jeong M.-J., Yoo J. (2007). Snail Is Required for Transforming Growth Factor-Beta-Induced Epithelial-Mesenchymal Transition by Activating PI3 Kinase/Akt Signal Pathway. Biochem. Biophys. Res. Commun..

[17] Hong K., Lou L., Gupta S., Ribeiro-Neto F., Altschuler D.L. (2008). A Novel Epac-Rap-PP2A Signaling Module Controls CAMP-Dependent Akt Regulation. J. Biol. Chem..

[18] Lee Y.J., Han H.J. (2009). Troglitazone Ameliorates High Glucose-Induced EMT and Dysfunction of SGLTs through PI3K/Akt, GSK-3β, Snail1, and β-Catenin in Renal Proximal Tubule Cells. Am. J. Physiol. Ren. Physiol..

[19] Bienz M. (2005). β-Catenin: A Pivot between Cell Adhesion and Wnt Signalling. Curr. Biol..

[20] Yook J.I., Li X.-Y., Ota I., Fearon E.R., Weiss S.J. (2005). Wnt-Dependent Regulation of the E-Cadherin Repressor Snail. J. Biol. Chem..

[21] Prokop J.W., Liu Y., Milsted A., Peng H., Rauscher F.J. (2013). A Method for in Silico Identification of SNAIL/SLUG DNA Binding Potentials to the E-Box Sequence Using Molecular Dynamics and Evolutionary Conserved Amino Acids. J. Mol. Model..

[22] Feng X., Qin H., Shi Q., Zhang Y., Zhou F., Wu H., Ding S., Niu Z., Lu Y., Shen P. (2014). Chrysin Attenuates Inflammation by Regulating M1/M2 Status via Activating PPARgamma. Biochem. Pharmacol..

[23] Jiang Y., Gong F.-L., Zhao G.-B., Li J. (2014). Chrysin Suppressed Inflammatory Responses and the Inducible Nitric Oxide Synthase Pathway after Spinal Cord Injury in Rats. Int. J. Mol. Sci..

[24] Anand K.V., Mohamed Jaabir M.S., Thomas P.A., Geraldine P. (2012). Protective Role of Chrysin against Oxidative Stress in D-Galactose-Induced Aging in an Experimental Rat Model. Geriatr. Gerontol. Int..

[25] Rashid S., Ali N., Nafees S., Ahmad S.T., Arjumand W., Hasan S.K., Sultana S. (2013). Alleviation of Doxorubicin-Induced Nephrotoxicity and Hepatotoxicity by Chrysin in Wistar Rats. Toxicol. Mech. Methods.

[26] Pingili R.B., Pawar A.K., Challa S.R., Kodali T., Koppula S., Toleti V. (2019). A Comprehensive Review on Hepatoprotective and Nephroprotective Activities of Chrysin against Various Drugs and Toxic Agents. Chem. Biol. Interact..

[27] Kang M.-K., Park S.-H., Choi Y.-J., Shin D., Kang Y.-H. (2015). Chrysin Inhibits Diabetic Renal Tubulointerstitial Fibrosis through Blocking Epithelial to Mesenchymal Transition. J. Mol. Med..

[28] Lee E.-J., Kang M.-K., Kim D.Y., Kim Y.-H., Oh H., Kang Y.-H. (2018). Chrysin Inhibits Advanced Glycation End Products-Induced Kidney Fibrosis in Renal Mesangial Cells and Diabetic Kidneys. Nutrients.

[29] Cohen D.J., Loertcher R., Rubin M.F., Tilney N.L., Carpenter C.B., Strom T.B. (1984). Cyclosporine: A New Immunosuppressive Agent for Organ Transplantation. Ann. Intern. Med..

[30] Shih W., Hines W.H., Neilson E.G. (1988). Effects of Cyclosporin A on the Development of Immune-Mediated Interstitial Nephritis. Kidney Int..

[31] Bennett W.M., DeMattos A., Meyer M.M., Andoh T., Barry J.M. (1996). Chronic Cyclosporine Nephropathy: The Achilles’ Heel of Immunosuppressive Therapy. Kidney Int..

[32] Klintmalm G., Sundelin B., Bohman S.-O., Wilczek H. (1984). Interstitial Fibrosis in Renal Allografts after 12 to 46 Months of Cyclosporin Treatment: Beneficial Effect of Low Doses in Early Post-Transplantation Period. Lancet.

[33] Ruiz P., Kolbeck P.C., Scroggs M.W., Sanfilippo F. (1988). Associations between Cyclosporine Therapy and Interstitial Fibrosis in Renal Allograft Biopsies. Transplantation.

[34] Jacobson S.H., Jaremko G., Duraj F.F., Wilczek H.E. (1996). Renal Fibrosis in Cyclosporin A-Treated Renal Allograft Recipients: Morphological Findings in Relation to Renal Hemodynamics. Transpl. Int..

[35] Shehata M., Cope G.H., Johnson T.S., Raftery A.T., El Nahas A.M. (1995). Cyclosporine Enhances the Expression of TGF-β in the Juxtaglomerular Cells of the Rat Kidney. Kidney Int..

[36] Islam M., Burke J.F., McGowan T.A., Zhu Y., Dunn S.R., McCue P., Kanalas J., Sharma K. (2001). Effect of Anti-Transforming Growth Factor-Βbgr; Antibodies in Cyclosporine-Induced Renal Dysfunction. Kidney Int..

[37] Liu Q., Ye J., Yu L., Dong X., Feng J., Xiong Y., Gu X., Li S. (2017). Klotho Mitigates Cyclosporine A (CsA)-Induced Epithelial–Mesenchymal Transition (EMT) and Renal Fibrosis in Rats. Int. Urol. Nephrol..

[38] McMorrow T., Gaffney M.M., Slattery C., Campbell E., Ryan M.P. (2005). Cyclosporine A Induced Epithelial–Mesenchymal Transition in Human Renal Proximal Tubular Epithelial Cells. Nephrol. Dial. Transplant..

[39] Slattery C., Campbell E., McMorrow T., Ryan M.P. (2005). Cyclosporine A-Induced Renal Fibrosis: A Role for Epithelial-Mesenchymal Transition. Am. J. Pathol..

[40] Kuruş M., Eşrefoğlu M., Bay A., Öztürk F. (2005). Protective Effect of Oral L-Arginine Supplementation on Cyclosporine Induced Nephropathy in Rats. Int. Urol. Nephrol..

[41] Ateşşahin A., Çeribaşı A.O., Yılmaz S. (2007). Lycopene, a Carotenoid, Attenuates Cyclosporine-Induced Renal Dysfunction and Oxidative Stress in Rats. Basic Clin. Pharmacol. Toxicol..

[42] Diegelmann R.F., Evans M.C. (2004). Wound Healing: An Overview of Acute, Fibrotic and Delayed Healing. Front. Biosci. J. Virtual Libr..

[43] Yao J., Jiang M., Zhang Y., Liu X., Du Q., Feng G. (2016). Chrysin Alleviates Allergic Inflammation and Airway Remodeling in a Murine Model of Chronic Asthma. Int. Immunopharmacol..

[44] Balta C., Herman H., Boldura O.M., Gasca I., Rosu M., Ardelean A., Hermenean A. (2015). Chrysin Attenuates Liver Fibrosis and Hepatic Stellate Cell Activation through TGF-β/Smad Signaling Pathway. Chem. Biol. Interact..

[45] Ali B.H., Al Za M., Adham S.A., Yasin J., Nemmar A., Schupp N. (2016). Therapeutic Effect of Chrysin on Adenine-Induced Chronic Kidney Disease in Rats. Cell. Physiol. Biochem..

[46] Sereno J., Rodrigues-Santos P., Vala H., Rocha-Pereira P., Alves R., Fernandes J., Santos-Silva A., Carvalho E., Teixeira F., Reis F. (2014). Transition from Cyclosporine-Induced Renal Dysfunction to Nephrotoxicity in an in Vivo Rat Model. Int. J. Mol. Sci..

[47] Esposito C., Fornoni A., Cornacchia F., Bellotti N., Fasoli G., Foschi A., Mazzucchelli I., Mazzullo T., Semeraro L., Dal Canton A. (2000). Cyclosporine Induces Different Responses in Human Epithelial, Endothelial and Fibroblast Cell Cultures. Kidney Int..

[48] Marconi G.D., Fonticoli L., Rajan T.S., Pierdomenico S.D., Trubiani O., Pizzicannella J., Diomede F. (2021). Epithelial-Mesenchymal Transition (EMT): The Type-2 EMT in Wound Healing, Tissue Regeneration and Organ Fibrosis. Cells.

[49] Kalluri R., Neilson E.G. (2003). Epithelial-Mesenchymal Transition and Its Implications for Fibrosis. J. Clin. Investig..

[50] Kasai H., Allen J.T., Mason R.M., Kamimura T., Zhang Z. (2005). TGF-Β1 Induces Human Alveolar Epithelial to Mesenchymal Cell Transition (EMT). Respir. Res..

[51] Yang B., Huang J., Xiang T., Yin X., Luo X., Huang J., Luo F., Li H., Li H., Ren G. (2014). Chrysin Inhibits Metastatic Potential of Human Triple-Negative Breast Cancer Cells by Modulating Matrix Metalloproteinase-10, Epithelial to Mesenchymal Transition, and PI3K/Akt Signaling Pathway. J. Appl. Toxicol..

[52] Katsuno Y., Lamouille S., Derynck R. (2013). TGF-β Signaling and Epithelial–Mesenchymal Transition in Cancer Progression. Curr. Opin. Oncol..

[53] Cabezas F., Farfán P., Marzolo M.-P. (2019). Participation of the SMAD2/3 Signalling Pathway in the down Regulation of Megalin/LRP2 by Transforming Growth Factor Beta (TGF-SS1). PLoS ONE.

[54] Akool E.-S., Doller A., Babelova A., Tsalastra W., Moreth K., Schaefer L., Pfeilschifter J., Eberhardt W. (2008). Molecular Mechanisms of TGFβ Receptor-Triggered Signaling Cascades Rapidly Induced by the Calcineurin Inhibitors Cyclosporin A and FK506. J. Immunol..

[55] Cho W., Kim Y., Kim J., Park S., Park D., Kim B.-C., Jeoung D., Kim Y.-M., Choe J. (2015). Suppressor of Cytokine Signaling 1 Is a Positive Regulator of TGF-β–Induced Prostaglandin Production in Human Follicular Dendritic Cell–like Cells. J. Immunol..

[56] Cano A., Perez-Moreno M.A., Rodrigo I., Locascio A., Blanco M.J., del Barrio M.G., Portillo F., Nieto M.A. (2000). The Transcription Factor Snail Controls Epithelial-Mesenchymal Transitions by Repressing E-Cadherin Expression. Nat. Cell Biol..

[57] Carver E.A., Jiang R., Lan Y., Oram K.F., Gridley T. (2001). The Mouse Snail Gene Encodes a Key Regulator of the Epithelial-Mesenchymal Transition. Mol. Cell. Biol..

[58] Batlle E., Sancho E., Franci C., Dominguez D., Monfar M., Baulida J., Garcia De Herreros A. (2000). The Transcription Factor Snail Is a Repressor of E-Cadherin Gene Expression in Epithelial Tumour Cells. Nat. Cell Biol..

[59] Medici D., Hay E.D., Olsen B.R., Bronner-Fraser M. (2008). Snail and Slug Promote Epithelial-Mesenchymal Transition through β-Catenin–T-Cell Factor-4-Dependent Expression of Transforming Growth Factor-Β3. Mol. Biol. Cell.

[60] Masszi A., Fan L., Rosivall L., McCulloch C.A., Rotstein O.D., Mucsi I., Kapus A. (2004). Integrity of Cell-Cell Contacts Is a Critical Regulator of TGF-Β1-Induced Epithelial-to-Myofibroblast Transition: Role for β-Catenin. Am. J. Pathol..

[61] Kang H.R., Lee C.G., Homer R.J., Elias J.A. (2007). Semaphorin 7A Plays a Critical Role in TGF-β1–Induced Pulmonary Fibrosis. J. Exp. Med..

[62] Caraci F., Gili E., Calafiore M., Failla M., La Rosa C., Crimi N., Sortino M.A., Nicoletti F., Copani A., Vancheri C. (2008). TGF-Β1 Targets the GSK-3β/β-Catenin Pathway via ERK Activation in the Transition of Human Lung Fibroblasts into Myofibroblasts. Pharmacol. Res..

[63] Peng J., Ramesh G., Sun L., Dong Z. (2012). Impaired Wound Healing in Hypoxic Renal Tubular Cells: Roles of Hypoxia-Inducible Factor-1 and Glycogen Synthase Kinase 3β/β-Catenin Signaling. J. Pharmacol. Exp. Ther..

[64] Chen Y., Luo Q., Xiong Z., Liang W., Chen L., Xiong Z. (2012). Telmisartan Counteracts TGF-Β1 Induced Epithelial-to-Mesenchymal Transition via PPAR-γ in Human Proximal Tubule Epithelial Cells. Int. J. Clin. Exp. Pathol..

[65] Hatanaka H., Koizumi N., Okumura N., Kay E.P., Mizuhara E., Hamuro J., Kinoshita S. (2012). Epithelial-Mesenchymal Transition-Like Phenotypic Changes of Retinal Pigment Epithelium Induced by TGF-β Are Prevented by PPAR-γ Agonists. Investig. Ophthalmol. Vis. Sci..

[66] Rani N., Bharti S., Bhatia J., Tomar A., Nag T.C., Ray R., Arya D.S. (2015). Inhibition of TGF-β by a Novel PPAR-γ Agonist, Chrysin, Salvages β-Receptor Stimulated Myocardial Injury in Rats through MAPKs-Dependent Mechanism. Nutr. Metab..

[67] Wang Y., Pang L., Zhang Y., Lin J., Zhou H. (2019). Fenofibrate Improved Interstitial Fibrosis of Renal Allograft through Inhibited Epithelial-Mesenchymal Transition Induced by Oxidative Stress. Oxid. Med. Cell. Longev..

[68] Lu Q., Wang W.-W., Zhang M.-Z., Ma Z.-X., Qiu X.-R., Shen M., Yin X.-X. (2019). ROS Induces Epithelial-Mesenchymal Transition via the TGF-Β1/PI3K/Akt/MTOR Pathway in Diabetic Nephropathy. Exp. Ther. Med..

[69] Zha D., Wu S., Gao P., Wu X. (2019). Telmisartan Attenuates Uric Acid-Induced Epithelial-Mesenchymal Transition in Renal Tubular Cells. BioMed. Res. Int..

[70] Wang Z., Li Y., Sarkar F.H. (2010). Signaling Mechanism(s) of Reactive Oxygen Species in Epithelial-Mesenchymal Transition Reminiscent of Cancer Stem Cells in Tumor Progression. Curr. Stem Cell Res. Ther..

[71] Samarghandian S., Farkhondeh T., Azimi-Nezhad M. (2017). Protective Effects of Chrysin Against Drugs and Toxic Agents. Dose Response.

[72] Kucukler S., Benzer F., Yildirim S., Gur C., Kandemir F.M., Bengu A.S., Ayna A., Caglayan C., Dortbudak M.B. (2021). Protective Effects of Chrysin Against Oxidative Stress and Inflammation Induced by Lead Acetate in Rat Kidneys: A Biochemical and Histopathological Approach. Biol. Trace Elem. Res..

[73] Sassi A., Boubaker J., Loussaief A., Jomaa K., Ghedira K., Chekir-Ghedira L. (2021). Protective Effect of Chrysin, a Dietary Flavone against Genotoxic and Oxidative Damage Induced by Mitomycin C in Balb/C Mice. Nutr. Cancer.

[74] Higgins D.F., Kimura K., Bernhardt W.M., Shrimanker N., Akai Y., Hohenstein B., Saito Y., Johnson R.S., Kretzler M., Cohen C.D. (2007). Hypoxia Promotes Fibrogenesis in Vivo via HIF-1 Stimulation of Epithelial-to-Mesenchymal Transition. J. Clin. Investig..

[75] Sun S., Ning X., Zhang Y., Lu Y., Nie Y., Han S., Liu L., Du R., Xia L., He L. (2009). Hypoxia-Inducible Factor-1α Induces Twist Expression in Tubular Epithelial Cells Subjected to Hypoxia, Leading to Epithelial-to-Mesenchymal Transition. Kidney Int..

[76] Fu B., Xue J., Li Z., Shi X., Jiang B.-H., Fang J. (2007). Chrysin Inhibits Expression of Hypoxia-Inducible Factor-1α through Reducing Hypoxia-Inducible Factor-1α Stability and Inhibiting Its Protein Synthesis. Mol. Cancer Ther..

[77] Dieperink H., Leyssac P.P., Starklint H., Kemp E. (1988). Long-Term Cyclosporin Nephrotoxicity in the Rat: Effects on Renal Function and Morphology. Nephrol. Dial. Transplant..

[78] Wang X., Morris M.E. (2008). Pharmacokinetics and Bioavailability of the Flavonoid 7,8-Benzoflavone in Rats. J. Pharm. Sci..

[79] Sunilkumar S., Ford S.M. (2019). Elevated Glucose Concentration in Culture Media Decreases Membrane Trafficking of SGLT2 in LLC-PK1 Cells via a CAMP/PKA-Dependent Pathway. Am. J. Physiol. Cell Physiol..

[80] Denoon T., Sunilkumar S., Ford S.M. (2020). Acetoacetate Enhances Oxidative Metabolism and Response to Toxicants of Cultured Kidney Cells. Toxicol. Lett..

[81] Vittal R., Horowitz J.C., Moore B.B., Zhang H., Martinez F.J., Toews G.B., Standiford T.J., Thannickal V.J. (2005). Modulation of Prosurvival Signaling in Fibroblasts by a Protein Kinase Inhibitor Protects against Fibrotic Tissue Injury. Am. J. Pathol..

