# Supplementary figures and images for: Chrysin Ameliorates Cyclosporine-A-Induced Renal Fibrosis by Inhibiting TGF-β1-Induced Epithelial–Mesenchymal Transition
Source: Int J Mol Sci. 2021 Sep 23;22(19):10252. doi: 10.3390/ijms221910252 (PMC8508845; doi:10.3390/ijms221910252)

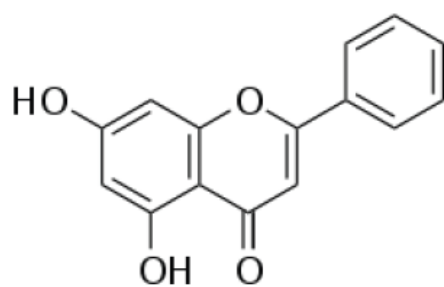

Chrysin

**Figure S1.** Structure of chrysin

Supplement: Supplementary file 1 [file ijms-22-10252-s001.zip › ijms-1352483-supplementary.pdf]
